# Electronic Cigarette Use and the Risk of Cardiovascular Diseases

**DOI:** 10.3389/fcvm.2022.879726

**Published:** 2022-04-07

**Authors:** Jorge Espinoza-Derout, Xuesi M. Shao, Candice J. Lao, Kamrul M. Hasan, Juan Carlos Rivera, Maria C. Jordan, Valentina Echeverria, Kenneth P. Roos, Amiya P. Sinha-Hikim, Theodore C. Friedman

**Affiliations:** ^1^Division of Endocrinology, Metabolism and Molecular Medicine, Department of Internal Medicine, Charles R. Drew University of Medicine and Science, Los Angeles, CA, United States; ^2^David Geffen School of Medicine, University of California, Los Angeles, Los Angeles, CA, United States; ^3^Research and Development Service, Bay Pines VA Healthcare System, Bay Pines, FL, United States; ^4^Laboratorio de Neurobiología, Facultad de Ciencias de la Salud, Universidad San Sebastián, Concepción, Chile; ^5^Friends Research Institute, Cerritos, CA, United States

**Keywords:** electronic cigarettes, nicotine, heart failure, cardiovascular disease, atherosclerosis

## Abstract

Electronic cigarettes or e-cigarettes are the most frequently used tobacco product among adolescents. Despite the widespread use of e-cigarettes and the known detrimental cardiac consequences of nicotine, the effects of e-cigarettes on the cardiovascular system are not well-known. Several *in vitro* and *in vivo* studies delineating the mechanisms of the impact of e-cigarettes on the cardiovascular system have been published. These include mechanisms associated with nicotine or other components of the aerosol or thermal degradation products of e-cigarettes. The increased hyperlipidemia, sympathetic dominance, endothelial dysfunction, DNA damage, and macrophage activation are prominent effects of e-cigarettes. Additionally, oxidative stress and inflammation are unifying mechanisms at many levels of the cardiovascular impairment induced by e-cigarette exposure. This review outlines the contribution of e-cigarettes in the development of cardiovascular diseases and their molecular underpinnings.

## Introduction

Tobacco consumption has been present in the Americas since prehistoric times (1). After tobacco was introduced from indigenous inhabitants of the Western hemisphere to Europeans through members of Columbus' crew (1), the effects of tobacco use have been a subject of scientific controversy. In the 16th century, the Spaniard physician and botanist Nicolás Monardes published a book explaining the therapeutic effects of tobacco use for dozens of health problems (2). Although in the 17th century, King James I of England spoke about the dangerous effects of tobacco (3), the systematic observations of the negative health effects of tobacco took longer than expected. In the first part of the 20th century, pathologists observed a strong association between lung cancer and cigarette smoking (4). The rate of smoking peaked in the 60s, with about 42% of the adult population in the United States being tobacco smokers in 1965 (5). By 2019, however, the smoking rate among adults aged 18 years or older went down to 14.0% (6). Currently, cigarette smoking is still the leading cause of preventable death, contributing to chronic obstructive pulmonary disease, several types of cancer, diabetes, and cardiovascular disease (CVD) (7), and is an additive risk factor in COVID-19 (8).

Although the use of cigarettes has decreased in the last few years, addiction to nicotine has continued due to the introduction of electronic cigarettes or e-cigarettes to the market. In 2004, a Beijing-based company, Ruyan Group (Holdings) Ltd., China, patented and launched e-cigarettes (9) that delivered nicotine to users without burning tobacco (10). E-cigarettes are battery-powered devices that produce an aerosol generated by heating a solution (e-liquid) consisting of nicotine, glycerol, propylene glycol, and flavors. The first generation of e-cigarettes tried to mimic the experience of smoking conventional cigarettes. The later generation devices contain high-powered atomizers and use higher nicotine concentrations in the e-liquids, increasing the speed of delivery and yield of nicotine like conventional cigarettes (11). JUUL and other pod-mods use nicotine formulations derived from the nicotine salts in loose-leaf tobacco (12, 13). Nicotine salts in pod-mods such as JUUL reduce harshness and result in a satisfying experience even at high nicotine concentrations (14). Although e-cigarettes were initially marketed as a smoking cessation tool, they will likely lead to future conventional cigarette smoking in people that have never smoke (15, 16). Especially concerning is the effect of e-cigarettes on the youth, and it is noteworthy that 19.6% of high school students used e-cigarettes in 2020 (17). Smokers who use e-cigarettes in an attempt to stop smoking often end up using both products. These dual users have been found to have higher cardiovascular risk factors than single users (18).

Smoking is associated with 11% of cardiovascular deaths worldwide (19). Currently, heart disease is the leading cause of death in the United States. Atherosclerosis is a chronic inflammatory condition associated with the accumulation of lipids and fibrous elements in the arteries where inflammatory cells are recruited to the arterial walls. The effects of conventional cigarettes on the cardiovascular system have been extensively studied (20, 21). Although e-cigarettes have the potential to be less harmful than conventional cigarettes due to their reduced number of harmful chemicals, the precise toxicological and mechanistic data of the effects e-cigarettes have on the cardiovascular system remain to be elucidated. A cross-sectional analysis of cardiovascular symptoms showed that e-cigarette users have a higher risk of coronary heart disease, arrhythmia, chest pain, or palpitations (22).

One of the complexities of studying the cardiovascular effects of e-cigarettes is the large variety of devices and chemical compositions of e-liquids. Different e-cigarettes' devices may produce different chemical products by thermal degradation of e-cigarette liquids and differences in the size of fine particulate matter (PM_2.5_) or ultrafine particles (UFPs). The negative effects of PM_2.5_ and UFPs on the cardiovascular system are well-established (23, 24). In indoor studies, e-cigarettes produce PM_2.5_ and UFPs concentrations ~45 and 20 times higher, respectively, than recommended by the World Health Organization (25). Additionally, the heating temperature in e-cigarettes creates metal particles (copper, nickel, and silver) from the atomizer unit (26) that are delivered into the bloodstream through the lungs.

The literature on the consequences of second-hand e-cigarette vaping is limited. However, chemical components are partially exhaled by users of electronic cigarettes. Consequently, it is important to study the effect of second-hand e-cigarette aerosol on CVD (27). Furthermore, exhaled nicotine and other e-liquid components can deposit onto surfaces and subsequently negatively affect the health of those exposed (third-hand exposure). In addition, toxicological studies of the components of e-cigarettes such as glycerol, propylene glycol, and artificial flavors have been mainly tested *via* oral administration, but very few studies have investigated the effect of aerosolizing the content of e-cigarettes at a high temperature. It is also widely known that E-liquid thermal decomposition produces the breakdown of glycerol and propylene glycol into toxic aldehydes, acetaldehyde, acrolein, and formaldehyde (28, 29). Acrolein produces lipid peroxidation and modifies the component of high-density lipoprotein (HDL) Apolipoprorein-I (ApoA-I). This fact leads to speculation on the role of chronic e-cigarette consumption in the development of atherosclerosis (30). Additionally, acrolein produces vascular oxidative stress (31) and platelet activation, a risk factor for thrombotic vascular events (32). Fortunately, the concentrations of acrolein produced by e-cigarettes are likely to be too low to have effects of clinical relevance (33). Given that the concentration of the carbonyl compounds positively correlates with the voltage and temperature of e-cigarettes (34), tight monitoring of the acrolein production from e-cigarettes may be necessary.

**Figure 1:**
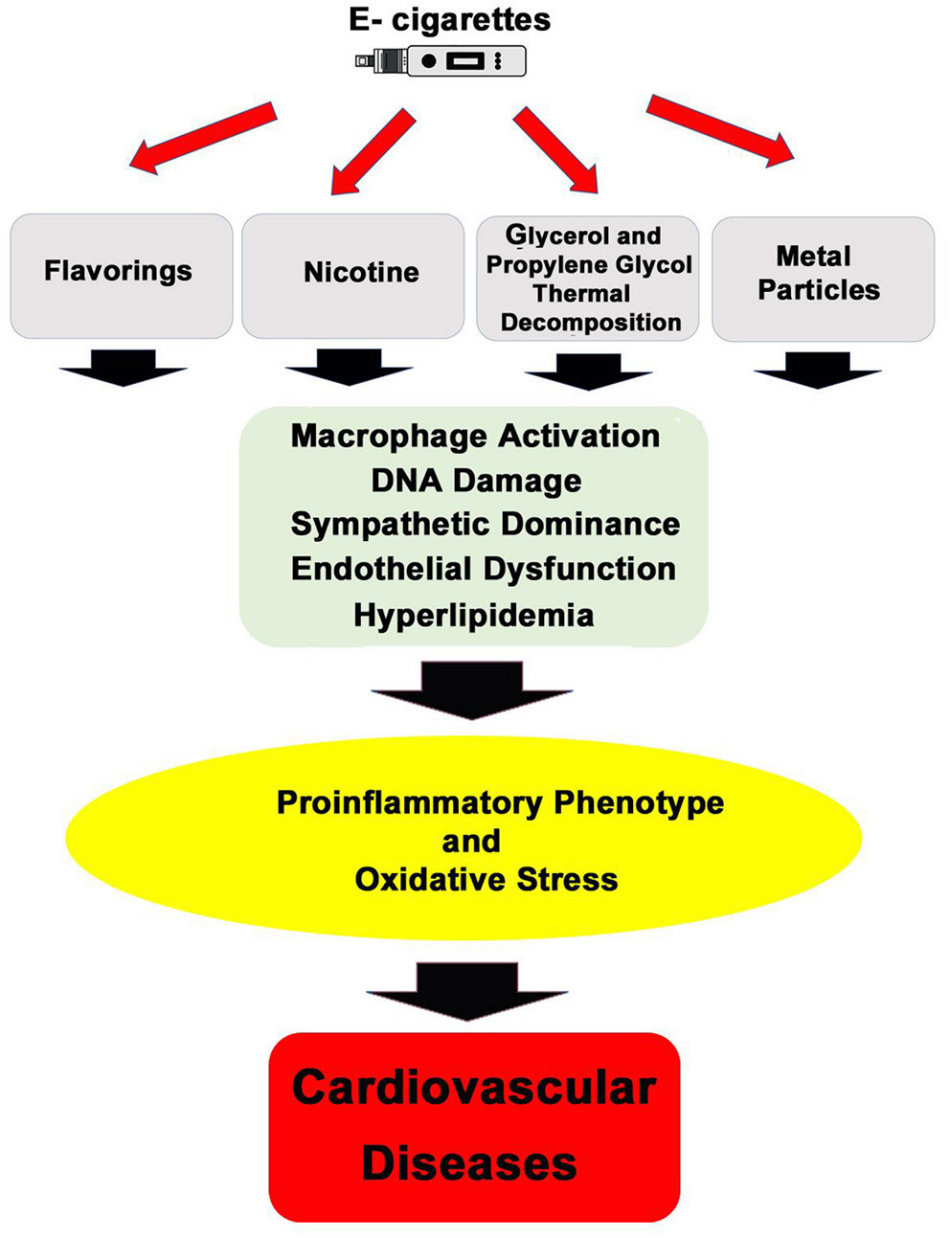
General mechanisms of action by which inhalation of e-cigarettes' aerosol can promote cardiovascular diseases.

Increasing the complexity of the toxicological analysis of e-cigarettes is the existence of more than 7,000 flavors for e-liquids in the market (35). Most of these thousands of flavors have been tested for oral ingestion safety; however, there is no complete data on their safety of the exposure to these components once heated and inhaled. For instance, experimental evidence shows that vanillin, cinnamaldehyde, eugenol, and acetylpyridine flavors induce nitric oxide and pro-inflammatory interleukins in endothelial cells (36), and vanilla custard e-vapor extract increased necrotic and apoptotic HL-1 cardiomyocyte cells (37). Adding to this complexity, e-cigarette users modify devices and solutions, which may further impact the toxicological characteristic of e-cigarettes (38). Figure 1 summarizes the proposed mechanisms of e-cigarettes' effects on the cardiovascular system.

## Nicotine

Nicotine is the most studied biologically active chemical present in e-cigarettes, and several of the cardiovascular effects of e-cigarettes have been attributed to this alkaloid from the tobacco plant (39). Nicotine is a highly addictive drug, having well-established effects on the metabolism (40–42) and cardiovascular system (7, 39, 43). Higher levels of nicotine in e-cigarettes have been associated with an increase in the frequency and intensity of combustible cigarette smoking (44).

The acute and chronic effects of nicotine differ as chronic exposure induces fast desensitization of the nicotine receptors. For instance, although nicotine in e-cigarettes can acutely increase blood pressure, chronic smoking has not been linked to higher blood pressure in most epidemiological studies (45). Nicotine has systemic hemodynamic effects that are mediated by the activation of the sympathetic nervous system. Thus, acute nicotine treatment can stimulate cardiac output by producing systemic vasoconstriction and increasing heart rate (45). Nicotine activates macrophages which infiltrate atherosclerotic lesions and release cytokines such as the TNF-1β and IL-1β that increase inflammation (46). The most common complication of atherosclerosis is the formation of a thrombus that leads to a stroke or a myocardial infarction. Nicotine has thrombogenic activity by activating platelets and coagulation cascades (20).

### Nicotinic Acetylcholine Receptors

Nicotine binds to the nicotinic acetylcholine receptors (nAChRs), which are integral membrane proteins that belong to the ligand-gated ion channel superfamily (47). Several nAChRs subunit combinations can form great diversity of functional receptors with a variety of specialized functions and properties depending on the cell type (48). Although the roles of nAChRs in synaptic transmission in the central (CNS) and peripheral nervous systems (PNS) are the most studied, nAChRs are present in several non-neuronal cells (49). In the cardiovascular system, vascular endothelial cells and smooth muscle cells (VSMC) express several subunits of nAChRs (50, 51). The activation of endothelial cells can lead to the release of vasoconstrictor substances. For instance, nicotine increases the release of endothelin-1 from human umbilical vein endothelial cells (52). Carotid arteries treated with nicotine show an impairment of endothelial-dependent relaxation associated with a decreased eNOS expression (53). In aortic smooth muscle cells, nicotine enhances insulin-induced mitogenesis through up-regulation of α7nAChR, a phenomenon associated with atherosclerosis (54). Additionally, nicotine has pro-angiogenic effects through the activation of α7nAChR (55). Nicotine produces arterial stiffness through extracellular matrix remodeling by upregulating matrix metalloproteinases (56). In the right ventricle, nicotine treatment lead to α7nAChR activation and fibroblast proliferation, collagen production, and extracellular matrix remodeling (57). Therefore, genetic or pharmacological inhibition of the α7nAChR rescues the effects of nicotine on right ventricular fibrosis (57).

### pH and Nicotine

E-cigarettes are formulated to have different pH levels, perhaps designed to increase their sensory impact (58). Traditional e-cigarette products use e-liquid with free-base nicotine while a new generation of e-cigarettes, the pod-mods, such as JUUL, use nicotine salts (12). The pH of the E-liquid is a function of the concentration of the free-base nicotine and the concentration of the nicotine salt. If the E-liquid contains free-base nicotine, the pH is high. If the E-liquid contains nicotine salt, the pH can be lower as a range from 5 to 7 (12). Previous studies have reported that the pH of e-liquids ranges from 8 to 10 for conventional e-cigarettes (59) and around 6 for JUUL (12, 60). Regarding the health effects, two aspects of e-liquid pH are often discussed: (1) Different sensory experiences. High pH e-cigarette aerosol appeared to be harsher, while lower pH close to physiological levels provides a more satisfying experience (13, 14). (2) Nicotine in an aqueous solution can exist in two main forms: monoprotonated [NicH^+^] and unprotonated [Nic] forms. The ratio of the concentrations of the unprotonated vs. protonated nicotine [Nic]/[NicH^+^] is a function of pH (the Henderson–Hasselbalch equation):


(1) $$\begin{array}{r} \text{pH}=\text{pKa}+\text{log}\left(\left[\text{Nic}\right]/\left[\text{Nic}{\text{H}}^{+}\right]\right) \end{array}$$

Where pKa is the logarithmic acid dissociation constant. Protonated nicotine is the ligand of nAChR (61). Theoretically, high protonated nicotine [NicH^+^] in the e-cigarette aerosol can have a greater impact on cells expressing the nAChRs in the respiratory tract (12). On the other hand, unprotonated nicotine [Nic] is lipophilic; thus, following inhalation, it would more readily diffuse across pulmonary cell membrane (12). High [Nic] can induce a rapid rising phase and higher peak concentrations in the arterial blood, and as a consequence, greater cardiovascular effects, increasing the risk of cardiovascular events such as cardiac arrhythmia, fluctuations of blood pressure and disruption of hemodynamic processes (62).

### Nicotine and Metabolic Syndrome

Smoking is a risk factor for insulin resistance in a dose-dependent manner (63). Smokers have lower glucose uptake and are more insulin resistant compared to nonsmokers (64). They also have higher plasma triglyceride (TG) and lower HDL-cholesterol levels (64). These findings on insulin resistance in smokers have been reproduced (65–71) and support other studies showing that insulin resistance can lead to both dyslipidemia (72) and endothelial dysfunction (73) seen in smokers. The degree of insulin resistance was also positively correlated to tobacco consumption (66), and, in long-term users of nicotine gum, to serum cotinine levels. Cotinine is a metabolite from nicotine; serum or urine levels of cotinine are considered to reflect the degree of nicotine use (63, 74). This implies nicotine as the causative agent of insulin resistance. In contrast to its effects on insulin sensitivity, cigarette smoking does not affect insulin secretion (64, 75, 76). Even acute smoking (one cigarette) impaired insulin sensitivity in healthy young men (66) and impaired glucose tolerance and insulin sensitivity in both smokers and non-smokers (77). Data from the Copenhagen male study indicated that only those smokers who have characteristic dyslipidemia associated with insulin resistance were at greatly increased CVD risk (78). Accordingly, with the effect of nicotine, e-cigarette users have higher fasting glucose levels than never users (79). In large-population-based studies, cigarette smoking was associated with an increased incidence of Type 2 diabetes mellitus (DM) (80) and metabolic syndrome [as defined by the National Cholesterol Education Program (81)]. A systematic meta-analysis confirmed the association between smoking and DM (82), and an editorial suggested that 12% of DM in the US is attributable to smoking (83). Cigarette smokers who were insulin sensitive did not display any abnormalities of lipoprotein metabolism (84). In contrast, cigarette smokers who were also insulin resistant had significantly higher plasma concentrations of TG and VLDL-cholesterol. Insulin resistance predicts the development of age-related diseases, including hypertension, stroke, coronary artery disease, cancer, and type 2 DM (85). Thus, it can be argued that a defect leading to increased CVD risk in smokers is insulin resistance and that the multiple adverse consequences associated with insulin resistance, including dyslipidemia and endothelial dysfunction, are responsible for the accelerated atherogenesis in these individuals (86).

## Impact of e-Cigarette Exposure on the Generation of Oxidative Stress and Inflammation

Atherosclerosis is a chronic inflammatory condition associated with the accumulation of lipids and fibrous elements in the arteries where inflammatory cells are recruited to the arterial walls. Increased production of reactive oxygen species (ROS) is a unifying mechanism for several risk factors that induce arteriosclerosis, endothelial cell dysfunction, and cardiac dysfunction (87–91). Increased ROS can produce activation of pro-apoptotic signaling resulting in cardiac remodeling and dysfunction (92). Mitochondria are both a major source of ROS and the primary target of ROS damage (93). Given that mitochondrial DNA (mtDNA) lacks protection from histones and has proximity to the source of ROS, it is very susceptible to oxidative alterations of nucleotides in the sequence of its coding regions (94). Thus, mtDNA mutations can lead to mitochondrial dysfunction and inefficient energy production of cardiac cells (95). Chronic ROS production results in the accumulation of mtDNA mutations, oxidized proteins, and lipids, leading to mitochondrial dysfunction and energy deficits in the heart (96).

E-cigarettes induce increased ROS *in vitro* and *in vivo* in endothelial cells (97), which leads to DNA damage (97), mt DNA mutations (39), and lipid peroxidation, all of which indicate oxidative stress and ROS- mediated damage of cells. ROS can directly impair the nitric oxide (NO)-mediated vasorelaxation by quenching NO (98). Exposure to e-cigarettes aerosols for 12 weeks induced an inflammatory phenotype consisting in high levels of lipid peroxidation and mitochondrial DNA mutations in a nicotine-dependent manner (39). Recently, it has been reported an increase in both lipid peroxidation and inflammation induced by e-cigarettes were associated with heart fibrosis in rats (99).

The role of inflammation in the development of atherosclerosis (100) and heart failure (101) is well-established. Transcriptomic analysis of hearts exposed to e-cigarettes showed that mice exposed to e-cigarettes had dysregulation of signaling factors involved in inflammation, circadian rhythm regulation, and leukocyte extravasation (39). In primary microvascular endothelial cells, e-cigarettes, and conventional cigarettes decreased the expression of the tight junctional protein Zonula Occludens-1 (ZO-1), suggesting that they alter the blood-brain barrier integrity (102). This effect was also accompanied by the activation of Nuclear factor-erythroid factor 2-related factor 2 (Nrf2), a main cellular transcription factor of the oxidative stress response, and Platelet endothelial cell adhesion molecule 1 (PECAM-1), a pro-inflammatory adhesion molecule (102). Also, this study revealed an upregulation of the inflammatory proteins, Intercellular adhesion molecule-1 (ICAM-1), and Vascular cell adhesion protein 1 (VCAM-1) in the brain homogenates of mice exposed to e-cigarettes (102).

Platelets from healthy volunteers exposed to e-cigarettes aerosol show an increase in the expression of globular complement protein C1q receptor (gC1qR) and calreticulin cC1q receptor (cC1qR), two proteins that are associated with the atherosclerotic events, platelet activation and aggregation. In a randomized crossover trial, 25 tobacco smokers were exposed to sham vaping, e-cigarettes without nicotine, and e-cigarettes with nicotine. Plasma myeloperoxidase, an enzyme highly expressed in neutrophils and macrophages used as a marker of an inflammatory process (103), was increased after exposure to e-cigarettes with nicotine, but not in patients exposed to sham vaping or e-cigarettes without nicotine (43). Other studies have shown increased inflammatory and oxidative stress in non-smokers even when exposed to e-cigarettes without nicotine (104). These data suggest a clear effect of e-cigarettes with nicotine on the production of oxidative stress and inflammation; however, further work is needed to uncover the effects of the non-nicotine e-cigarette contributions to atherosclerosis.

## Blood Lipids

High levels of triglycerides and low levels of high-density lipoproteins (HDL) are risk factors for cardiovascular disease (105, 106). Nicotine promotes loss of body weight and the disturbance of lipoprotein metabolism through the secretion of catecholamines, such as norepinephrine. Catecholamine secretion favors the elevation of LDL and very low density lipoproteins (VLDL) and is also associated with decreased HDL levels (107–109). Higher levels of LDL and VLDL are also known risk factors for cardiovascular diseases (110–112). A health survey in Korean men showed significantly elevated triglyceride levels in dual users of conventional cigarettes and e-cigarettes compared to non-smokers (18). In addition, there was no significant difference in triglyceride levels between dual users and conventional cigarette-only smokers. However, another work has shown that e-cigarette users have higher triglycerides and lower HDL than never users (79). HDL cholesterol was significantly lower in both dual users and conventional cigarette only smokers compared to those who never have smoked (18). E-cigarette vapers had increased levels of LDL and VLDL compared to nonsmokers (113). Habitual e-cigarette users had increased oxidized LDL levels compared with non-user control individuals (114). Oxidized LDL can lead to atherosclerosis as it can contribute to the buildup of atherosclerotic plaques (20). In a gender-specific effect, a plasma lipidome analysis showed that female but not male e-cigarette users had decreased plasmalogens levels (115). Plasmalogens have a protective role against lipid peroxidation (116). Clinical trials, animal experiments, and surveys have shown associations between e-cigarette use and negative health outcomes concerning blood lipids and potential cardiovascular diseases; however, more clinical, animal, and epidemiological studies will be needed to establish causation with these negative health outcomes.

## Free Fatty Acids

Adipocyte dysfunction produces systemic inflammation, a pathogenic mechanism underlying the well-known associations between obesity, cardiovascular pathology, hypertension, and metabolic syndrome (117). Thus, adipose tissue, an important regulator of the cardiovascular system (118, 119), produces bioactive factors that regulate lipid levels and is involved in inflammation, oxidative stress, and insulin resistance (120). Epidemiological studies have shown that the combination of smoking and obesity results in a higher mortality risk (121). Additionally, smokers have a subclinical systemic inflammation with decreased adiponectin levels in plasma (122). The role of the adipose tissue on the effects of smoking in vascular pathology is highlighted by evidence showing that the epicardial adipose tissue and subcutaneous adipose tissue in smokers has higher levels of inflammatory adipokines, namely TNF-α and IL-6 than found in this tissue in non-smokers (123).

In cultured 3T3L1 adipocytes, nicotine-induced AMP-activated protein kinase (AMPK) phosphorylation, lipolysis, and oxidative stress in a concentration-dependent manner (124). Furthermore, the activation of nAChRs by nicotine stimulated AMPK activation led to the release of free fatty acids (FFAs) from rodent adipocytes (125–128). Additionally, systemic administration of nicotine produced lipolysis by inducing the release of catecholamines that bind to β-adrenergic receptors located in adipocytes (129).

In humans, cigarette smoking and obesity have been associated with increased levels of FFAs (130), which, in turn, are correlated with an increased risk for CVD (131, 132). FFAs have been broadly studied in their contribution to the induction of metabolic changes that lead to metabolic syndrome (133) and adverse cardiovascular outcomes (134). Increased FFAs produce a low inflammatory state that is characterized by infiltration and expansion of lymphocytes and macrophages, which produce pro-inflammatory cytokines that interfere with insulin signaling (135). FFAs are likely one of the key elements in ectopic lipid accumulation, lipotoxicity, mitochondrial dysfunction (136–138), and cardiomyopathy (132, 139). Improvements in whole-body insulin sensitivity can be obtained by pharmacological reduction of chronically elevated plasma FFA levels (140, 141). Our laboratory has shown that e-cigarettes produce increased plasma levels of FFA and intramyocardial lipid accumulation (39). Additionally, we have shown that inhibition of lipolysis using acipimox inhibited the hepatic metabolic changes induced by consuming a high-fat diet plus nicotine (142). Increased FFA promotes inflammation in adipose tissue through the activation of the toll-like receptor 4 (TLR4) signaling (118, 143). Our team found that e-cigarettes induce a cardiac inflammatory phenotype associated with cardiac dysfunction and atherosclerosis (39). Associated with this phenotype, we also found increased levels of serum FFA and oxidative stress (39). Therefore, we postulate that the nicotine present in e-cigarettes increases the levels of FFA and ROS, leading to atherosclerosis and cardiac dysfunction (39).

**Figure 2:**
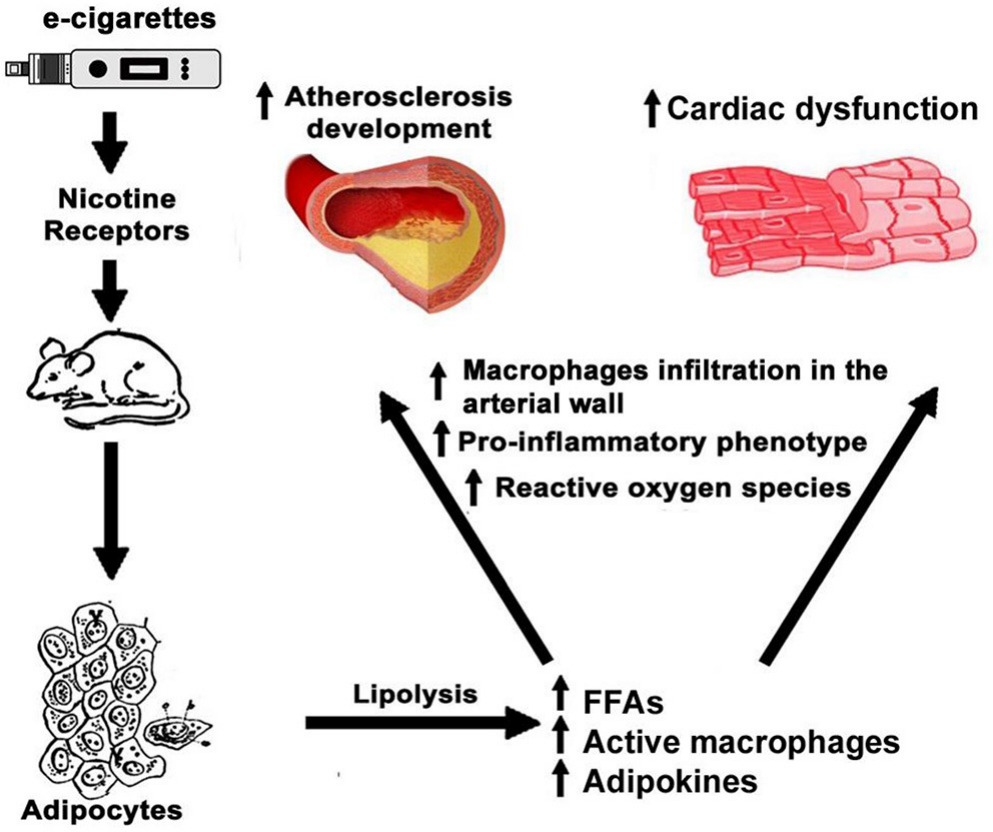
Effects of e-cigarettes on adipocytes and cardiovascular system. In adipocyte tissue, nicotine in e-cigarettes produces the release of FFA and adipokines leading to the activation of macrophages and an inflammatory phenotype that detrimentally affects the cardiovascular system function.

In a hyperlipidemic, low-density lipoprotein receptor null mouse model, nicotine stimulated macrophages to secrete inflammatory cytokines, creating a pro-inflammatory microenvironment in the sub-endothelium that increased the aortic lesion size by 2.5 times (46). Human monocytes are sensitive to cigarette smoking, NF-κB activation, and the production of pro-inflammatory cytokines such as IL-8 (144). After infiltration in the arterial wall, monocytes differentiate into macrophages and engulf oxidized low density lipoproteins (LDL) with the help of scavenger receptors creating foam cells, which secrete cytokines that, in turn, recruit more immune cells (145). In Apolipoprotein E (ApoE) null mice, e-cigarettes induced a cardiac inflammatory phenotype associated with increased serum levels of FFA and atherosclerosis (39). Together, this evidence suggests that the effects of nicotine and e-cigarettes on FFA, adipokines, and inflammatory cells are potent modulators of cardiovascular physiology. We depict a possible mechanism of these effects in Figure 2. Aside from the increase of FFA by e-cigarettes, the reported changes in adipose tissue by nicotine warrant further examination of the role of adipocyte tissue on the recruitment and activation of immune cells to the heart and vasculature.

## Sympathetic Dominance

Cardiac sympathetic activation is a risk factor for cardiovascular disease (146), and habitual e-cigarette users have an increased activation sympathetic system (114). Accordingly, e-cigarettes have been reported to produce sympathetic dominance in humans (114) and mouse models (147). Sympathetic and parasympathetic terminals innervate sinoatrial (SA) and atrioventricular (AV) nodes in the heart. In contrast, arteries and veins only receive sympathetic innervation (148). nAChRs are the key mediators of synaptic transmission in autonomic ganglia (148). Nicotine binding to nAChRs led to a release of adrenaline from the adrenal medulla and noradrenaline from postganglionic sympatric nerves, activating the sympathetic nervous system (7, 149). Blood vessels have α1 and β2 adrenoreceptors, which produce vasoconstriction and vasodilatation, respectively. Since the higher presence of α1 receptor in vessels, high adrenaline concentrations induce vasoconstriction through activation of α1 receptors (148). β1 adrenergic receptors are expressed in the heart, and their activation leads to increased cardiac contractility and heart rate (148). The chronic activation of the sympathetic system produces an increased cardiac overload and inflammation, leading to cardiac remodeling (150). Consistently with the relevance of this mechanism, β-blockers reduce heart failure mortality (151). Inhaling e-cigarettes (JUUL with nicotine) acutely increased sympathetic neural outflow in young, healthy non-smokers. In contrast, inhalation of a placebo e-cigarette without nicotine elicited no sympathetic dominance (152). Therefore, the sympathetic dominance produced by e-cigarettes is mainly produced by nicotine.

Sympathetic dominance activates the Splenocardiac axis. In this pro-inflammatory axis, the sympathetic stimulation of hematopoietic tissues increases circulating pro-inflammatory monocytes, increasing atherosclerosis and ischemic heart disease (153). The finding that e-cigarettes activated metabolic activity on spleen and blood vessel walls suggests the activation of the Splenocardiac axis (154).

Sympathetic activity plays a central role in the control of blood pressure. The acute effect of nicotine producing increased blood pressure has been well-researched through independent studies on nicotine and other cigarette studies (155–158). Inhalation of nicotine aerosol equivalent to cigarette smoking induces acute high magnitude irregular fluctuations of blood pressure in a pregnant rat model, with the arterial blood pressure being continuously measured (62). The irregular fluctuations of blood pressure primarily result from nicotine-induced cardiac arrhythmia (62) and probably can not be detected by the intermittent measurement with the conventional Korotkoff method. However, several epidemiological studies have found inconsistent results in comparing blood pressure levels among traditional cigarette smokers and non-smokers (159). E-cigarettes release nicotine and, after thermal degradation of propylene glycol and glycerin, release aldehydes (160), all of which can potentially cause an acute increase in blood pressure (161). As mentioned above, nicotine elevates blood pressure through the release of norepinephrine and epinephrine (162). The effects of aldehydes derived from the use of e-cigarettes are less studied, and the current potential effects and mechanisms are inferred from animal studies directly exposed to aldehydes. In rats, inhaled aerosol of acetaldehydes and propionaldehydes increased blood pressure by activating the sympathetic nervous system through the stimulation of the release of catecholamines (163). Previous studies have shown that aliphatic aldehydes, besides formaldehyde, had sympathomimetic activity. Aliphatic aldehydes most likely regulate blood pressure by the activation of the sympathetic system found in a study with anesthetized rats described above (163). More studies are needed to further understand the discrepancy between the effects of different types of aldehydes and the potential acute and chronic effects on blood pressure.

A comparison study found similar rates of acute increase in blood pressure when comparing e-cigarette and conventional cigarette use in smokers (164, 165). E-cigarette devices as the vape pod, JUUL, increased the blood pressure by 6 mm Hg acutely in comparison to nicotine-free e-cigarettes (152). Nevertheless, the authors have mentioned that the 6 mmHg of increase could be underestimated because the participants were not experienced e-cigarette users, which could be less nicotine absorption (152). Compared with nonsmokers, conventional cigarettes and e-cigarettes users have a similar pattern of increase in systolic blood pressure, with lesser effects in e-cigarette users (166). A small double-blinded clinical trial revealed that e-cigarettes with nicotine, but not without nicotine increased the peripheral systolic blood pressure and heart rate in humans (167). Conventional smokers with arterial hypertension that switched to e-cigarettes showed improvements in systolic and diastolic blood pressure (168). Three other acute comparison studies found that e-cigarette users had decreased blood pressure when compared to conventional cigarette users (169–171). Overall, a meta-analysis of the immediate effects of e-cigarettes found that acute use of nicotine e-cigarettes was associated with increased heart rate, systolic blood pressure, diastolic blood pressure (172). In an acute study, following 45 min of exposure to e-cigarette aerosol, blood pressure levels rose to similar levels of those who smoked tobacco cigarettes for 15 min, suggesting that a longer time is needed for e-cigarette exposure than conventional cigarette exposure on blood pressure (167). One of the acute studies also used carotid- pulse wave velocity to show that an increase of e-cigarette usage from 5 to 30 min increased arterial stiffness to a similar level to that of tobacco smokers (164).

A mouse study indicated little changes in blood pressure among mice that were exposed to filtered air, e-cigarettes, and conventional cigarettes for 8 months (147). A human study that lasted 3.5 years, found that there was no significant difference in long-term changes in blood pressure when comparing daily e-cigarette users and non-users (173).

Most of the acute comparison studies investigated the temporary increase of blood pressure levels after a short period of e-cigarettes use, most likely due to sympathetic nervous system activation. There are still discrepancies in these acute studies on whether e-cigarette use induced a lower elevation in blood pressure when compared to the use of conventional cigarettes. More data is necessary to fully understand the chronic implications of e-cigarettes use and the potential effects of the ingredients on blood pressure.

## Platelet Activation and Thrombogenesis

Platelet adhesion is a common trait of CVD (174). A whole-body e-cigarette mouse exposure protocol showed that e-cigarettes induce platelet activation with enhanced aggregation (169). Fine particulate matter also increased platelet adhesion and activation (175). Exposing mice to e-cigarettes for either 5 or 14 consecutive days increased the activation of platelets as well as shortened thrombosis after exposure to e-cigarettes (176).

Mice with short-term whole-body exposure to e-cigarettes developed a prothrombotic phenotype with hyperactive platelets and higher integrin and phosphatidylserine expression (176). E-cigarettes increased phosphorylated protein kinase B (Akt) and extracellular signal-regulated kinases (ERK), which are involved in platelet function (176, 177).

Conventional cigarettes and e-cigarettes produced a significant increase in platelet activation in non-smokers (178). However, there was also a lower increase of platelet aggregation following e-cigarette use than with conventional cigarette use (178). In a similar study comparing acute effects of conventional cigarette use and e-cigarette use, there was a similar increase in both groups of Nox2, a protein that regulates platelet-activation-associated thrombosis (165). *In vitro* studies of platelet exposure to e-cigarette aerosol also found increases in CD40 and P-selectin (175), which are markers for active platelets and thrombo-inflammation, respectively (161, 179). In *in vivo* studies, exposure to e-cigarettes for 5–7 days led to enhanced P-selectin levels after e-cigarette usage (176).

Clinical studies involving tobacco users with controlled conventional cigarette compared to e-cigarette with nicotine use showed acute increases in CD40 and P-selectin markers in conventional cigarette and e-cigarette users (165, 178, 180). Another study, looking at e-cigarette use with and without nicotine, found a similar increase of CD40 and P-selectin in users of e-cigarettes containing nicotine. In the groups that inhaled aerosol without nicotine, only CD40 increased (181). In summary, clinical and preclinical studies indicate that e-cigarettes consumption increases platelet aggregation, which can have a negative impact by potentiating cardiovascular events.

## Vascular Trauma and Coronary Vascular Disease

The effects of conventional cigarettes in vascular injury and CVD are well-established (7). Daily users of e-cigarettes have an increased risk factor for myocardial infarction (182). This risk appeared similar between e-cigarette and conventional cigarette smokers and was increased in users of both cigarettes and e-cigarettes (dual users) (183). The Framingham Heart Study showed a strong association between aortic stiffness and a higher incidence of cardiovascular events (184). Arterial stiffening leads to increased cardiovascular risk, including heart failure, myocardial infarction, and increased mortality. Hemodamicaly, arterial stiffening leads to increased blood pressure, cardiac workload, and decreased myocardial perfusion. Structural components of the arterial wall mainly determine arterial stiffening. Estimation of arterial stiffness is commonly measured by aortic-femoral pulse wave velocity (PWV), that is, the time that it takes for the arterial pulse to propagate from the carotid to the femoral artery. Several studies have shown that e-cigarettes increase PWV in humans (43, 164, 185). Mice exposed to e-cigarettes for 5 days a week for 8 months showed increased aortic arterial stiffness measured by PWV (147).

To differentiate the vascular effects of nicotine and carriers, a single-blind crossover design study was performed with patients exposed to vaping without nicotine, vaping with nicotine, and sham-vaping. Results from these clinical studies showed that nicotine from e-cigarettes reduced microvessel endothelial function, increased arterial stiffness, and triggered an increase in plasma myeloperoxidase (43). Nicotine-free e-cigarettes did not change microcirculatory function as well as arterial stiffness and oxidative stress markers (43). In healthy volunteers, two biomarkers for heightened vascular risk, microvesicles, and endothelial progenitor cells, were increased following exposure to e-cigarettes (180), and these effects were shown to be dependent on nicotine (181). *Ex vivo* experiments of wire tension myography and force transduction showed an increased thoracic aortic tension in response to vasoactive-inducing compounds in mice chronically exposed to e-cigarettes (147). In animal models, mice were exposed for 60 weeks to the e-cigarette with a concentration of nicotine from 0 to 24 mg/mL showed endothelial dysfunction and an increase in endothelial ROS, in addition to a thickening of the vessel wall were dependent on nicotine concentration (186). Experiments in mice showed that e-cigarettes produced uncoupling of eNOS, and peroxynitrite formation may lead to vascular endothelial dysfunction (187). Additionally, ROS causes endothelial dysfunction by directly quenching NO (98). On the other hand, flow-mediated dilation, a marker for the presence of subclinical atherosclerosis, was studied in conventional cigarettes smokers, e-cigarettes smokers, and non-smokers. Conventional cigarette smokers develop impairment of flow-mediated dilation compared to non-smokers, and electronic cigarette smokers have similar flow-mediated dilation as non-smokers. Therefore, this work suggests that the impairment of flow-mediated vasodilation may be nicotine-independent (188). An article on the beneficial effects of the switch from traditional cigarettes to e-cigarettes showed an early beneficial impact on endothelial function after this switch (189). The improvement in flow-mediated dilatation was mainly observed in females and non-dual users with little effect in dual users (189). However, one potential limitation of this study is the lack of a non-smoker control group.

Pulse wave analysis (PWA) is a technique commonly used to determine systemic arterial stiffness. The primary outcome derived from PWA is the augmentation index (AIx), which is normalized to the heart rate. Several groups have shown an increase in the AIx after e-cigarette and conventional cigarette use (43, 167, 185). Chaumont et al. (43) associated these hemodynamic parameters and ROS changes to nicotine without the influence of the non-nicotine components in the e-cigarettes.

The two most studied mechanisms for the effect of conventional cigarettes on vasculature are oxidative stress and the promotion of a pro-inflammatory state (7). In endothelial cells, e-cigarette and conventional cigarette extracts produce DNA damage, ROS generation, and apoptosis; however, cytotoxic effects were blunted by antioxidants (97), suggesting a pivotal role of ROS. These effects were greater in conventional cigarettes than e-cigarettes (97). E-cigarette exposure caused endothelial dysfunction through ROS in human endothelial cells derived from induced pluripotent stem cells (iPSC), and their conditioned media induced a pro-inflammatory state in macrophages (190). These effects were potentiated in cinnamon-flavored products (190).

In human umbilical vein endothelial (HUVEC) cells, e-cigarettes produced complement deposition, a phenomenon present in atherosclerotic lesions (191). This inflammatory phenomenon was related to a reduction of metabolic activity of endothelial cells (191). Consistently, sections of aortic root stained with Oil Red O from ApoE KO mice exposed to e-cigarettes aerosol, producing blood cotinine levels equivalent to that found in heavy smokers, showed increased development of atherosclerotic lesions (192). Additionally, e-cigarettes induced an innate immune response associated with aberrant neutrophilic activation in human airway samples (193). A similar model of e-cigarette induced atherosclerosis showed that damaged mitochondrial DNA in circulating blood may produce the increase of Toll-like receptor 9 (TLR9), leading to increased pro-inflammatory cytokines and macrophages activation (194). Additionally, pharmacological inhibition of TLR9 can attenuate the e-cigarettes exacerbated atherosclerosis in ApoE KO mice (194). Therefore, growing literature shows evidence for e-cigarette-induced vascular injury.

## Cardiac Function

Smoking is a significant predictor of mortality in people with heart failure (118, 195). The components of e-cigarettes or their heat-produced derivatives have been shown to have an effect on cardiac physiology. For instance, formaldehyde decreased in left ventricle end-systolic pressure and cardiac output (196), and acetaldehyde produced myocardial mitochondrial damage (197). Nicotine aerosol inhalation induces acute cardiac arrhythmia where sinoatrial (SA) block, sinus arrest, atrioventricular (A-V) block and supraventricular escape rhythm were demonstrated by ECG analysis in rats suggesting a disturbance of parasympathetic and sympathetic cardiac control mediated by the nAChRs (62). Mouse models for e-cigarette exposure with pharmacokinetics resembling human e-cigarettes have been developed (192). In the ApoE KO mice model exposed to 12 weeks of e-cigarettes with cotinine levels similar to the range of heavy smokers, RNA-seq analysis revealed dysregulation of inflammatory pathways (39). Additionally, M-Mode echocardiographic analysis showed a decreased left ventricular fractional shortening (LV%FS), ejection fraction (LVEF), and velocity of circumferential fiber shortening (VCF) in mice exposed to e-cigarettes with nicotine (39). However, exposure to e-cigarettes without nicotine did not affect cardiac function (39). Such changes in cardiac function were associated with increased ultrastructural abnormalities indicative of cardiac dysfunction and MDA generation, a marker of oxidative stress but without hypertrophy (39). These changes were not associated with changes in the gross morphology of the heart. Recently, C57BL/6J mice on an HFD were exposed to e-cigarettes in the presence (2.4% nicotine) or absence (0% nicotine) of nicotine and saline aerosol for 12 weeks (198). Indeed, we found a decrease in LV%FS, LVEF, and VCF coupled with ultrastructural abnormalities indicative of cardiomyopathy in mice treated with e-cigarette (2.4% nicotine) compared to e-cigarette (0% nicotine) or saline exposed mice (198). Therefore, nicotine seems to be necessary for the induction of systolic dysfunction induced by e-cigarettes in this mouse model (39). More extended exposure to e-cigarettes of 60 weeks, led to the development of cardiac left ventricular hypertrophy with an increased systemic vascular resistance (186). Accordingly, another study showed that mice exposed to e-cigarettes for 3 months had increased systolic blood pressure and diastolic blood pressure, associated with cardiac and renal fibrosis and a systemic inflammatory state (199). Recently, a similar phenotype produced by e-cigarettes in rats showed fibrosis, inflammation, and oxidative stress, but with cardiac hypertrophy (99).

## Cardiovascular Studies of Smokers Using e-Cigarettes as a Tool for Smoking Cessation

A meta-analysis of cardiovascular outcomes of smokers switching from traditional cigarettes to e-cigarettes did not show any improvement in stroke, myocardial infarction, or coronary heart disease outcomes (200). However, this work showed a reduction in adverse respiratory effects in smokers who switched to e-cigarettes (200). Additionally, larger studies have shown that the smokers who do not halt smoking often continue using both conventional cigarettes and e-cigarettes (dual users) (18). Compared with those who only smoke conventional cigarettes, dual users have a higher cardiovascular risk (201).

## Heat-Not-Burn Tobacco Cigarettes and CVD Safety

The tobacco industry's most recent development is a product deemed as a heat-not-burn (HnB) tobacco cigarettes (202, 203). These products claim to “heat” tobacco (rolled cast leaf sheets of tobacco soaked in propylene glycol) to temperatures around 350 Celsius rather than burn it at roughly 600°C. This creates an aerosol that contains nicotine which can be inhaled by users and is promoted to be less toxic and harmful than e-cigarette aerosol or smoke from combusted cigarettes (204). Phillip Morris International (PMI) is spearheading this new “technology.” Their new product (I-Quit-Ordinary-Smoking (IQOS) is being marketed to 1 day replace conventional cigarettes (205). Before this product gains traction, studies on its acute and long-term CVD safety are urgently needed.

## Discussion: Conclusions and Future

Although the public endorses the perception that e-cigarettes are safe (206), their long-term effects on human health will take a long time to be fully elucidated. In the last several years, e-cigarettes have been progressively regulated. The tobacco prevention act gave authority to the Food and Drug Administration (FDA) to regulate the production, distribution, and marketing of e-cigarettes. The evolving nature of the presented data in this work calls for more regulation, which would include additional safety testing for new flavors and devices that continue to emerge. Studies comparing the CVD effect of e-cigarettes vs. conventional cigarettes and especially those who use e-cigarettes to attempt to quit conventional cigarettes are urgently needed.

The past several years have been important in establishing the effects of e-cigarettes on CVD. Although the reduction of conventional cigarettes harm by substituting them with e-cigarettes remains under discussion, the molecular mechanism of e-cigarette effects in the cardiovascular system clearly is emerging. The proposed mechanisms for the effects of e-cigarettes on CVD are shared with common diseases that affect the general population. Mechanisms such as oxidative stress, inflammation, lipid accumulation, and sympathetic dominance are commonly present in non-smoking patients with atherosclerosis and diabetic cardiomyopathy (207). This leads to increased concern for people with metabolic or cardiovascular comorbidities that use e-cigarettes. Therefore, these shared mechanisms call for future studies investigating the impact of e-cigarettes on the cardiovascular disease on susceptible populations or representative animal models of these conditions. Additionally, the variety of flavors and delivery systems that change in different vendors will need to be studied. Future discussions in the field may be informed by the different effects of nicotine on the variety of nAChRs and target organs.

Nicotine has a dichotomic role as the main substance in harm-reduction products and a harmful substance for the CVS. Therefore, the solution for stopping the deleterious effects of smoking is nicotine cessation and not shifting the source of nicotine delivery. Alternatively, e-cigarettes may be an imperfect, comparatively safer alternative to conventional cigarettes that could be used as a cessation tool; however, the efficiency of this intervention is still uncertain. As a requirement for a specific e-cigarette to be designed as safer than conventional cigarettes, they need to be compared to conventional cigarettes. E-cigarette devices and e-liquids keep changing, and the safety information obtained today may not be valid in a few years. Likely, the scientific discussion that started centuries ago about smoking and health for Dr. Monardes (2) is certain to continue with e-cigarettes. We hope that consensus will come in a shorter time than the one established for conventional cigarettes.

## Author Contributions

JE-D and TF conceived and planned the work. JE-D, XMS, and TF wrote the manuscript. JE-D, XMS, CL, KH, JR, MJ, VE, KR, AS-H, and TF contributed to interpreting the literature and editing the manuscript. All authors contributed to the article and approved the submitted version.

## Funding

This work was supported by the NIH grants: NIGMS (SC2GM135127), NIMHD (S21MD000103), NHLBI (R01HL135623), NIDA (R42DA044788), vouchers from the NIH Accelerating Excellence in Translational Science (AXIS) (U54MD007598), and NIDA (R25DA050723). California TRDRP grant (28CP-0040), and DODCDMRP grant (PR190942) to TF. VE was funded by an ANID-FONDECYT (1190264).

## Conflict of Interest

The authors declare that the research was conducted in the absence of any commercial or financial relationships that could be construed as a potential conflict of interest.

## Publisher's Note

All claims expressed in this article are solely those of the authors and do not necessarily represent those of their affiliated organizations, or those of the publisher, the editors and the reviewers. Any product that may be evaluated in this article, or claim that may be made by its manufacturer, is not guaranteed or endorsed by the publisher.
